# Dataset on the integrated downdraft gasifier and multi integrated gas cleaner system (IGCS) for municipal solid waste (MSW)

**DOI:** 10.1016/j.dib.2020.105521

**Published:** 2020-04-19

**Authors:** Apri Wiyono, Nugroho Agung Pambudi, Miftah Hijriawan, Indra Mamad Gandidi, Asep Setiadi Husen, Purnawan

**Affiliations:** a Department of Mechanical Engineering Education, Faculty of Technology and Vocational Education, Universitas Pendidikan Indonesia, Jl. Dr. Setia Budi 299, Sukasari, Kota Bandung 40154, Indonesia; b Department of Mechanical Engineering Education, Teacher Training and Education Faculty, Sebelas Maret University, J. Ahmad Yani 200A, Surakarta 57161, Indonesia

**Keywords:** Biomass, Gasifier downdraft, Multi integrated gas cleaner system (IGCS), Municipal Solid Waste (MSW)

## Abstract

This experiment uses the Municipal Solid Waste (MSW) from households and traditional markets as feed materials in the Integrated Downdraft Gasifier and Multi Integrated Gas Cleaner System (IGCS). The IGCS consist of cyclone, rectangular venturi scrubber, and rotary separator. The data from the experiment show the gasification characteristics such as temperature, Low Heating Value (LHV) and tar content. The parameter consists of Air Fuel ration (AFR) at 0.48, 0.5, 0.54 scrubbing water discharge at 1.26, 2.62, 3.33 l/min, and 0.9 rotary separator using suction speed at 0.9, 3.4, 4.4 m/s, respectively. The data also show the power output of the plant and energy balance of the system. This data can be used as reference for the further development of Integrated Downdraft Gasifier and Multi-Integrated Gas Cleaner systems.

Specifications Table

**Table:** 

Subject	Renewable Energy, Sustainability and the Environment
Specific subject area	Biomass processing experiments using integrated systems
Type of data	Table Chart Figure
How data were acquired	This is an experimental study with the development of a downdraft gasifier and Integrated Gas Cleaner System (IGCS) consisting of cyclones, rectangular venturi scrubs, and rotary separators. The data were acquired using instruments such as bomb calorimeter AC 500, 5E-MAG6700 Proximate Analyzer, ultimate analyzer, anemometer, thermocouple-K, water pump Panasonic GP-129JXK, digital stopwatch, digital scale CAS-SW 1A CAP 30 kg, and digital multimeter Fluke 179 True-RMS. Furthermore, the analysis software used was Microsoft excel.
Data format	Raw and Analysed Data
Parameters for data collection	The experiment was carried out with air-fuel ratio parameters at 0.48, 0.5, 0.54, scrub water discharge at 1.26, 2.62, 3.33 l/min, and 0.9 rotary separator using suction speed at 0.9, 3.4, 4.4 m/s.
Description of data collection	Data were collected from a biomass power plant to produce gas with a Low Heating Value (LHV) greater than 2500 kJ/Nm^3^ and tar 100 mg/Nm^3^
Data source location	Bandung, West Java, Indonesia
Data accessibility	With the article
Related research article	A. Wiyono, I. M. Gandidi, A. S. Husen, Purnawan and N. A. Pambudi, “Design, development and testing of integrated downdraft gasifier and multi IGCS system of MSW for remote areas,” *Case Stud. Therm. Eng.*, p. 100,612, 2020.

## Value of the data

• This data presents the experimental results of Municipal Solid Waste (MSW) processing using a downdraft gasifier and multi Integrated Gas Cleaner System (IGCS) on several variables to show the gasification characteristics, calorific value, and tar content obtained.
• The data can be used as a reference in determining the experimental design of similar subsequent research and development.
• The method used in obtaining data, acts as a reference used to determine further design of the experiment to obtain better results
• With the same method and data, different materials can be used to optimize the system.

## 1 Data description

**Fig. 1:**
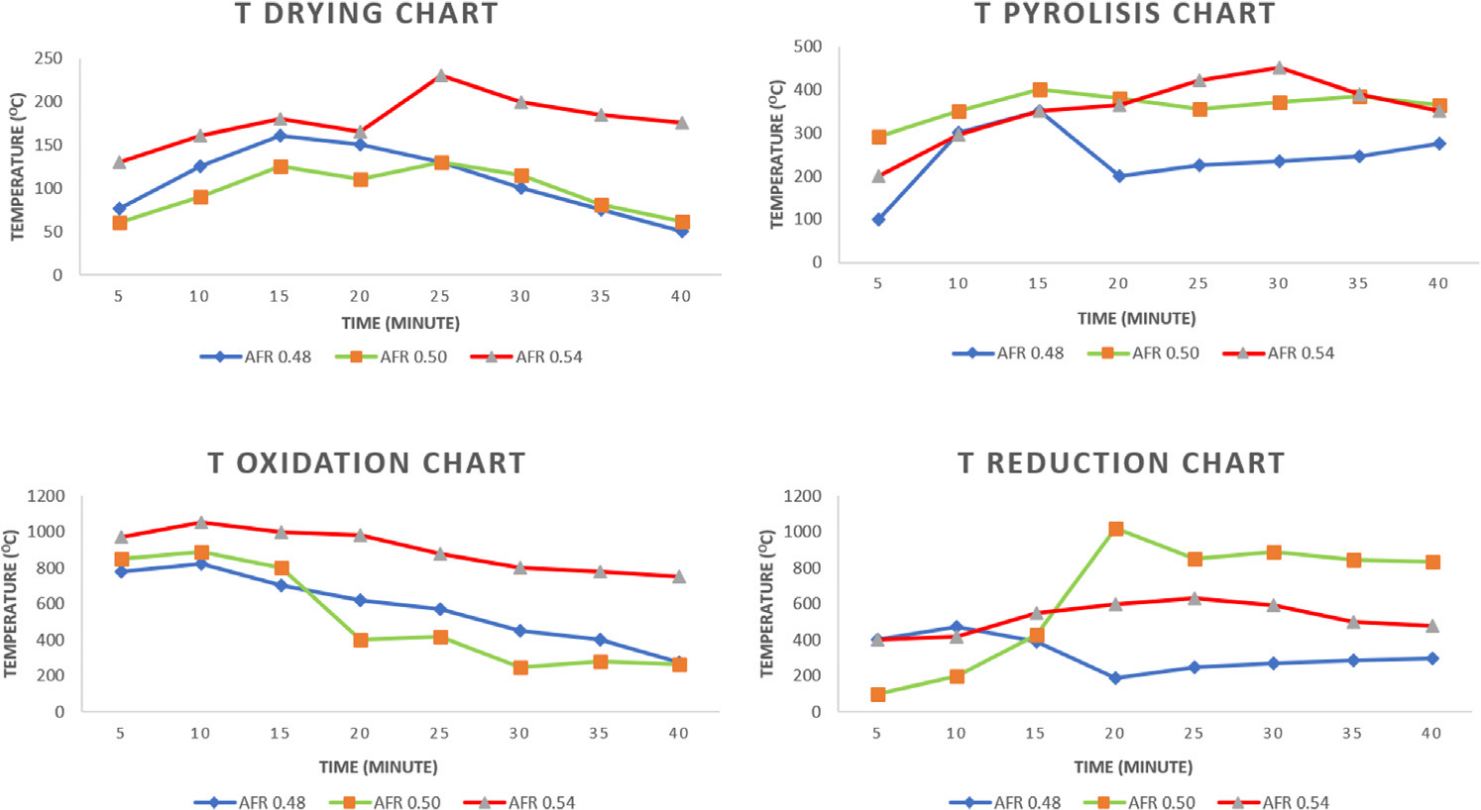
Temperature profile of the drying, pyrolysis, oxidation and reduction zones.

**Fig. 2:**
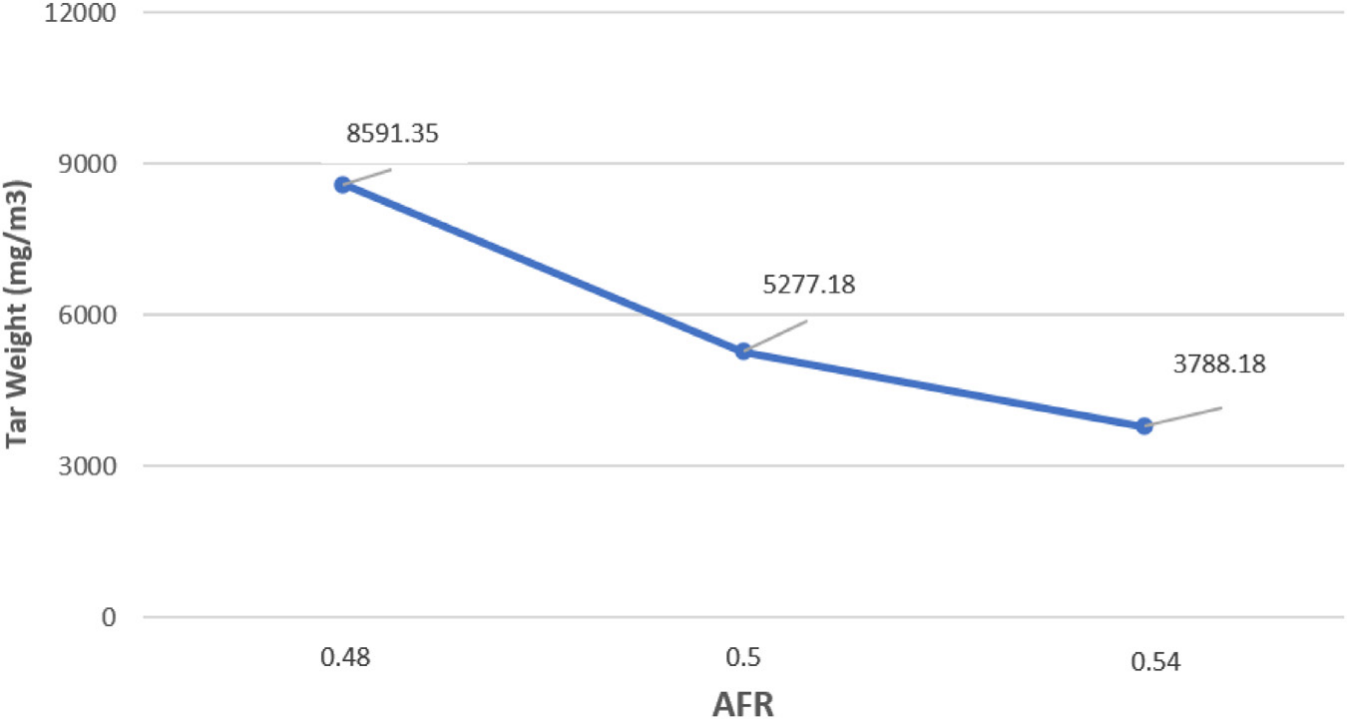
Effect of AFR on tar formation of the organic MSW gasification.

**Fig. 3:**
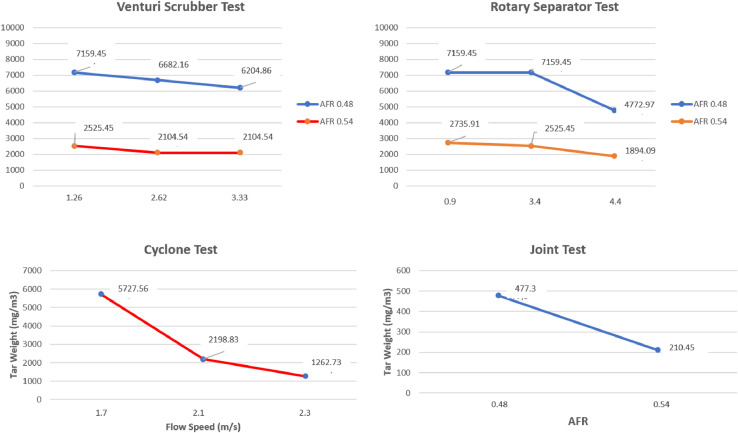
Tar weight on Integrated Gas Cleaning Systems (IGCS).

**Fig. 4:**
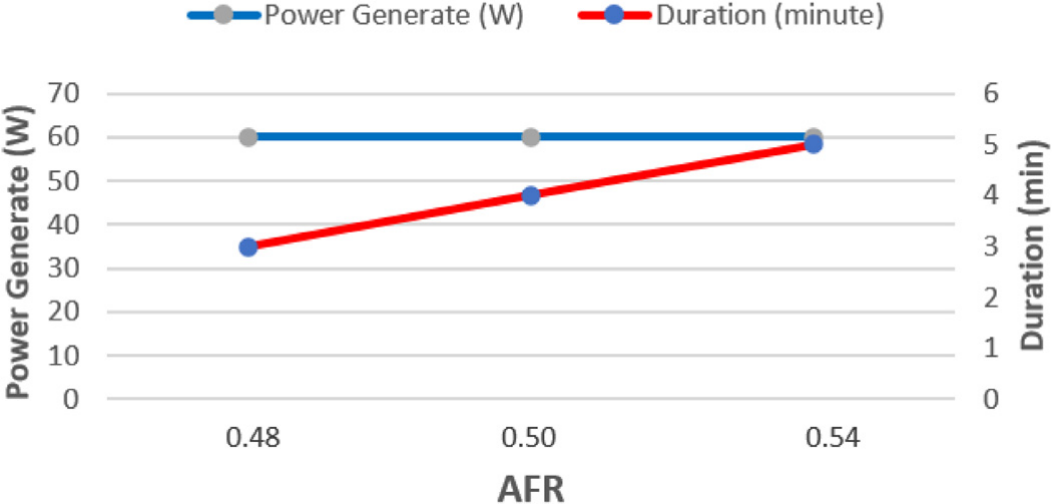
The power output of AFR variation of multi IGCS gasifier.

The data presented were obtained from design and experimental examination of all components of the gasification plant which included gasifier reactors and gas cleaners. The system consists of a cyclone, a venturi scrubber and a rotary separator designed to produce gas with a Low Heating Value (LHV) greater than 2500 kJ/Nm3 and tar 100 mg/Nm3 [1,2]. Table 1 shows the Air Fuel Ratio (AFR) calculated from various air mass flow rate used in organic Municipal Solid Waste (MSW). Fig. 1 then shows the temperature profile of drying, pyrolysis, oxidation and the reduction zones of each AFR. It also shows the gasification performance characteristics of the feed. Fig. 2 shows the effect of AFR on the formation of tar, while Fig. 3 shows the Tar weight in multi IGCS system. Fig. 4 shows the comparison of power output in various AFR. In addition, Tables 2 and 3 show the incoming and outcoming energy balance on the system.

**Table 1:** AFR gasification system.

Mass (kg)	Blower voltage (V)	Air mass flow rate (kg/s)	Gasification duration (minute)	AFR
1.5	100	0.0007	17.3	0.48
1.5	175	0.0008	15.2	0.5
1.5	250	0.0009	14.5	0.54

**Fig. 5:**
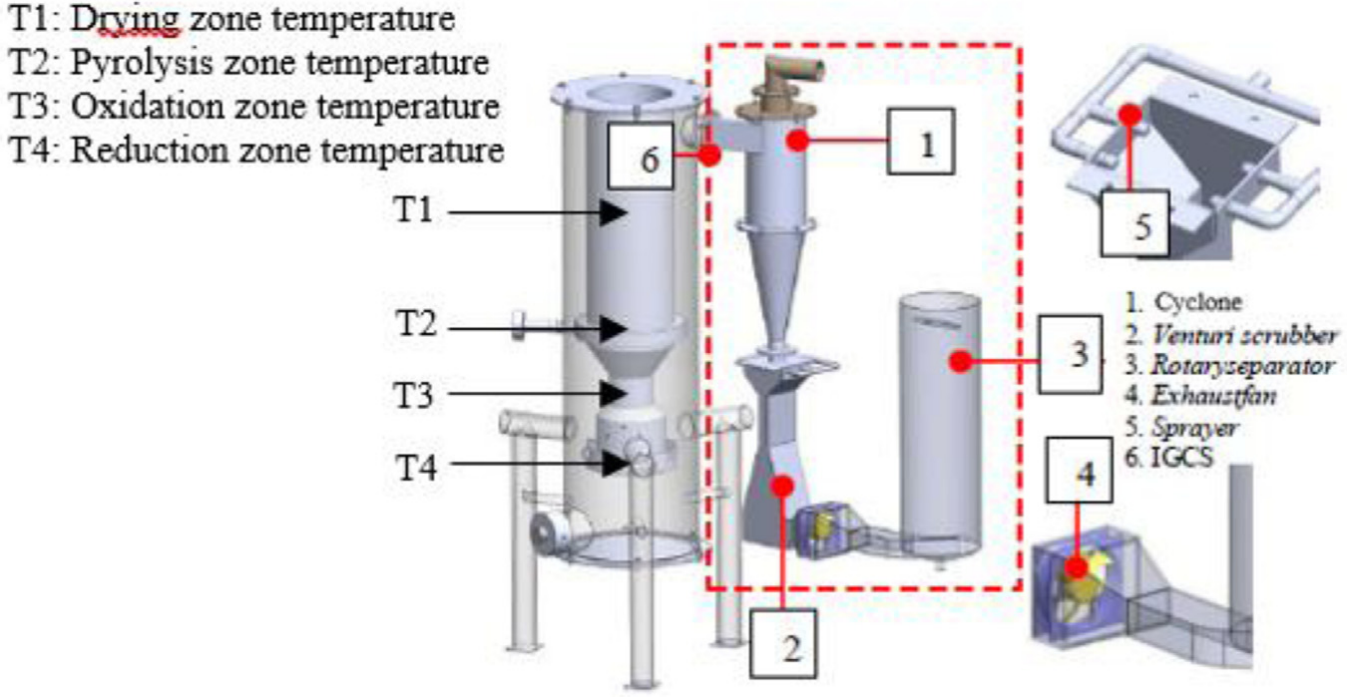
Downdraft Gasifier integrated IGCS Unit System Apparatus.

**Table 2:** Incoming energy balance.

AFR	Incoming energy balance (kJ/s)				
	Organic MSW	Char	Air	Electricity	Total input
0.48	20.690	28.736	0.211	1.189	50.825
0.5	20.690	28.736	0.256	1.189	50.870
0.54	20.690	28.736	0.278	1.189	50.892

**Table 3:** Outgoing energy balance.

AFR	Outgoing energy balance (kJ/s)			
	Producer gas	Heat loss	Total ouput	Energy Difference
0.48	25.870	0.139	26.010	24.816
0.5	31.186	0.187	31.373	19.497
0.54	33.686	0.237	33.923	16.969

## 2 Experimental design, materials, and methods

### 2.1 Characteristics of organic municipal solid waste (MSW)

Organic Municipal Solid Waste (MSW) such as wood, leaves, paper, food, vegetables, and fruits were collected from various households and traditional markets in Bandung, Indonesia. The raw materials are dried using solar heat for 3–4 h, it then examined to obtain proximate, ultimate, and heating values as shown in Table 4.

**Table 4:** Proximate, ultimate, and heating value of Organic MSW.

	Run 1	R 2	R 3	*Av*
Bomb Calorimeter				
Caloric value (cal/g)	3300	3300.2	3300.5	3300.2
Ultimate Analysis				
Nitrogen (wt,%, adb)	0.29	0.28	0.29	0.28
Carbon (wt,%, adb)	35.03	36.03	35.12	35.4
Hydrogen (wt,%, adb)	5.46	5.46	5.49	5.47
Sulfur (wt,%, adb)	0.12	0.12	0.12	0.12
Proximate Analysis				
Moisture (wt,%, adb)	7.82	7.8	7.82	7.81
Volatile (wt,%, adb)	57.66	57.66	57.65	57.7
Fixed Carbon (wt,%, adb)	13.91	13.92	13.91	13.9
Ash (wt,%, adb)	20.61	20.61	20.62	20.6

### 2.2 Design and manufacture

#### 2.2.1 Reactor design

The design of the Imbert downdraft gasifier is based on the specific gasification rate, also called the fireplace load (Bh), where N indicates that the gas volume is calculated under normal atmospheric temperature and pressure. The maximum of Bh in the Imbert gasifier is 0.9 for continuous operating conditions with a minimum range of 0.3–0.35 [3,4].

The value of the fireplace load is determined by using the following formula [3]

(1) $$B_{h}=\frac{V_{g}}{A_{t}}$$

The gasifier diameter (D) is determined by using the following formula

(2) $$D={\left(\frac{1.27\;FCR}{SGR}\right)}^{0.5}$$

where FCR is the level of fuel consumption (kg/hour)

In addition, the gasifier height (H) is determined by using the formula

(3) $$H=\frac{SGR\;.\;t}{\rho_{f}}$$

where SGR is the specific gasification speed (kg /hm2), t is the batch operating time (h), and ρf is the compatibility of raw material (kg/m^3^)

Five tuyeres are used to supply air requirements with the diameter (d) obtained by using the formula

(4) $$d={\left(\frac{1.27\;x\;AFR}{v\;x\;Z}\right)}^{0.5}$$

where v is the air inlet speed in the tuyere (m/s) and Z is the number of tuyeres [5].

#### 2.2.2 Cyclone separator design

The preliminary data used to design a cyclone are shown in Table 5.

**Table 5:** First data for designing cyclone [6].

No.	Specified parameters	Value
1	Solid density (ρ_p_)	389 kg/m^3^
2	Producer gas density (ρ_f_)	0.6179 kg/m^3^
3	Producer gas viscosity at 300 °C	296.404 × 10^−7^ kg/ms
4	Ash particle diameter (D_pi_)	856 µm
5	Cyclone inlet size	75 mm
6	Efficiency	98%

The cyclone inlet width passed by the organic MSW in the form of particles to the cyclone (Dp, th) with a theoretical efficiency of *V*_*in*_ = 10 m/s, is calculated using the formula

(5) $$d_{p,th\;}\sqrt{\frac{9\;\cdot \;\mu \;\cdot \;B_{C}}{\pi \;\cdot \;N_{s\;}\cdot \;V_{in\;}\cdot \;\left(\rho_{p}\;-\;\rho_{f}\right)\;}}$$

The number of gas cycles in the cyclone (Ns) with *V*_*in*_ = 10 m/s is estimated to obtain Ns = 2.5 times. The theoretical efficiency values are calculated using the formula (with *D_pi_* as the particle size from the cutting) [7].

(6) $$\eta_{th}=\frac{D_{pi}}{D_{p,th}}$$

### 2.3 Venturi scrubber design

The flow rate used to determine the velocity of producer gas in the venturi with *V* = 10 m/s is determined by the formula [8]

(7) $${\dot{m}}_{wv(in)}water\;vapour=\left[Q_{m(in)}.\theta_{H_{2}O\left(in\right)}\right]\frac{MW_{wv}}{V_{mole}}$$

(8) $${\dot{m}}_{a(in)}dry\;air=\left[Q_{m(in)}.1\theta_{H_{2}O\left(in\right)}\right]\frac{MW_{a}}{V_{mole}}$$

The amount of water production in the venturi scrubber is determined by the formula [7]

(9) $${\dot{m}}_{wv(evap)}{\dot{m}}_{wv(out)}\cdot {\dot{m}}_{wv(in)}$$

(10) $$Q_{wv\left(evap\right)}=\;\frac{{\dot{m}}_{wv\left(evap\right)}}{\rho_{H_{2}O}}$$

The next step is to determine the venturi size design, with the initial parameters searched using the standard deviation with the formula

(11) $$\sigma =\frac{d_{84}}{d_{50}}$$

The specification of d_cut_ based on ηd alloy with efficiency collection for particle size of 5 µm is shown in Table 6.

**Table 6:** The efficiency collection [8].

Range (µm)	Mass fraction	Eff. collection needed	Eff. collection fractional
0–1	0.005	0.900	0.0045
1–2.5	0.195	0.950	0.185
2.5–4.5	0.400	0.980	0.392
4.5–7	0.300	0.990	0.297
12-Jul	0.080	1.000	0.080
>12	0.020	1.000	0.020
Eff. collection whole			0.979

Scrubber power, pressure decrease and water/L /G ratio are determined by the following formula [8]

(12) $$A={\left[\frac{1270\;\cdot \;\Delta P}{v^{2}\;\cdot \;\rho_{g\;\cdot \;\;{\left(L\;/\;G\right)}^{0,78}}}\right]}^{1\;/\;0,133}$$

### 2.4 Rotary separator design

This is designed based on the particle separation techniques using the centrifugal force and speed for each organic MSW ash particle as shown in the formula [7]

(13) $$d_{p}={\left[\frac{18\;\cdot \;V_{t}\;\cdot \;\mu }{g\left(\rho_{f}-\rho_{p}\right)}\right]}^{1\;/\;2}$$

Based on Table 7, the Vt value of 8.09 m /s, is used as a reference for selecting the exhaust fan to form a forced flow in the rotary separator system.

**Table 7:** Parameter of the preliminary data of rotary separator [6].

No.	Specified data	Value
1.	Solid Density (*ρ_p_*)	389 kg/m^3^
2.	Producer gas density at temperature 40 °C (*ρf*)	1.118 kg/m^3^
3.	The viscosity of producer gas at temperature 40 °C (*µ*)	190,736.10^7^ N/sm^2^
4.	The particle diameter of feedstocks ash (*D_pi_*)	1556 µm
5.	Size of *inlet separator* (*Hc*)	820 mm

### 2.5 Quality examination procedure

The experiments are carried out by varying the discharge blower set with regulators at 100 V, 175 V, and 250 V by setting multi IGCS on water discharge scrubbing at 1.26 l/min, 2.62 l/min, 3.33 l/min, with a variation of the rotary separator suction fan by 0.9 m/s, 3.4 m/s, 4.4 m/s.

## Declaration of Competing Interest

The authors declare that they have no known competing financial interests or personal relationships that could have appeared to influence the work reported in this paper.

## Appendix Supplementary materials

Image, application 1

Image, application 2
