# Supplementary material for: Dataset on the integrated downdraft gasifier and multi integrated gas cleaner system (IGCS) for municipal solid waste (MSW)
Source: Data Brief. 2020 Apr 19;30:105521. doi: 10.1016/j.dib.2020.105521 (PMC7215098; doi:10.1016/j.dib.2020.105521)
Supplement: Supplementary file 1 [file mmc1.docx]

Specification of instrument how data were acquired

Specification of bomb calorimeter


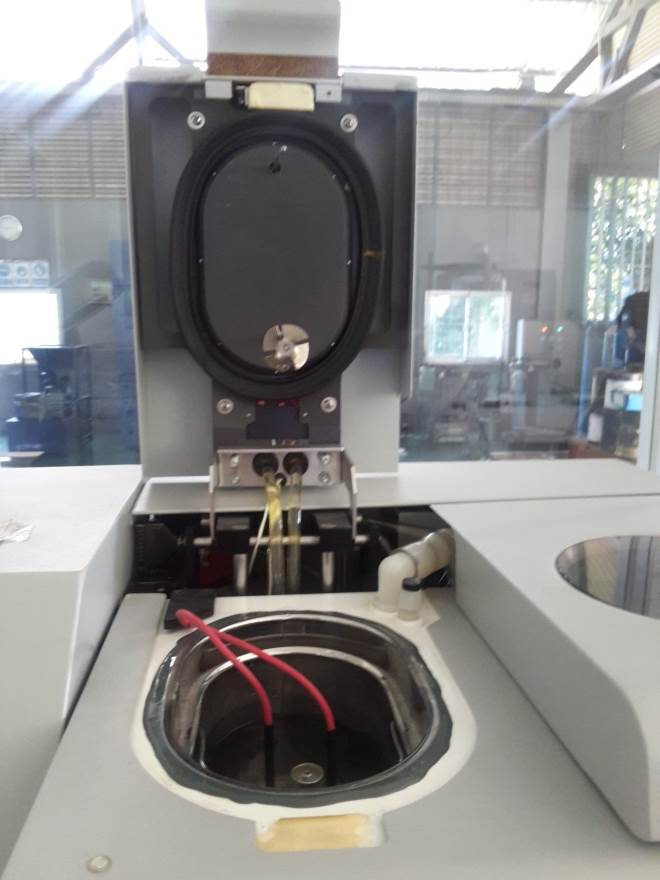

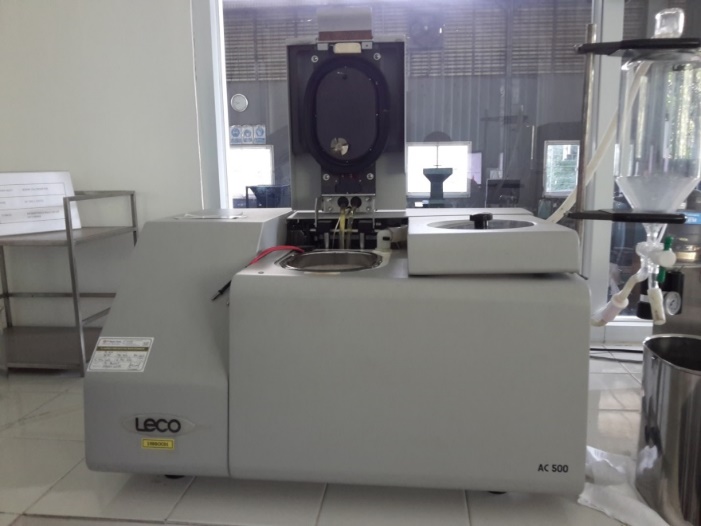


Specification of 5E-MAG6700 Proximate Analyzer


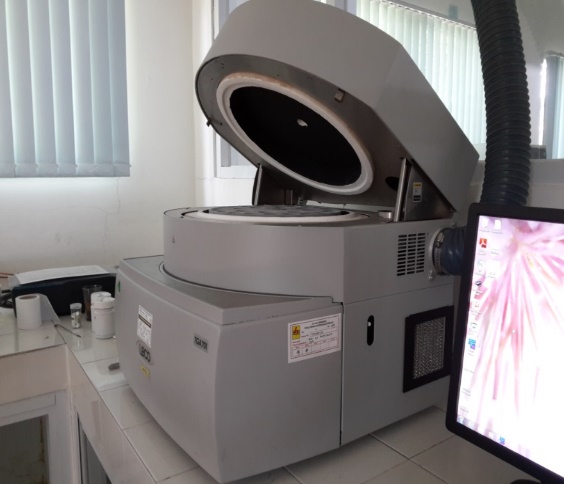

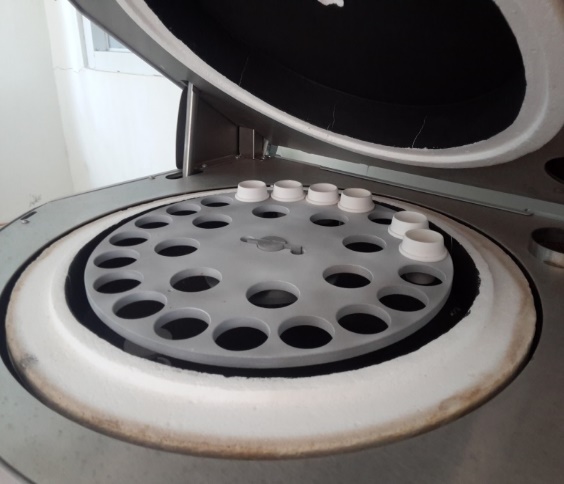


| Model | 5E-MAG6700 Proximate Analyzer - TGA | |
| --- | --- | --- |
| Conforms to Method | ASTM D3173/D3174/D3175/DD7582, ISO 17246, GB/T 30732, GB/T 212, ISO 18123/18122/18134 | |
| Max. Sample Loading | 19 | |
| Furnace | Dual furnace | |
| Analysis Time | ≤120mins for 19 samples | |
| Sample Mass | 0.8-1.2g recommended / up to 5g | |
| Temp. Range | Up to 1050℃ | |
| Temp. Control Precision | ± 2℃ | |
| Precision of Balance | 0.1mg | |
| Power Supply | Single phase, AC220V±10%, 50/60Hz, | Part I: ≤ 4 kW |
|  |  | Part II: ≤ 5 kW |
| Net Weight | Part I: 80kg | Part II: 50kg |
| Dimensions (L×W×H) | Part I: 550mm×580mm×890mm | Part II: 550mm×580mm×530mm |

Specification of ultimate analyzer


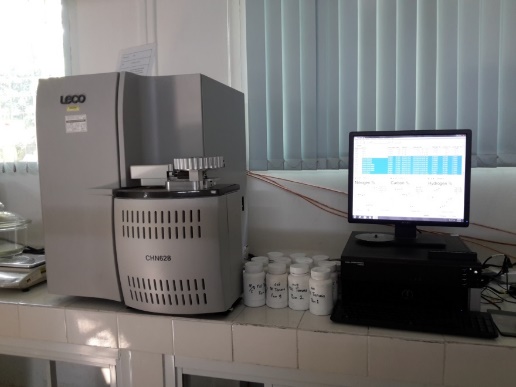

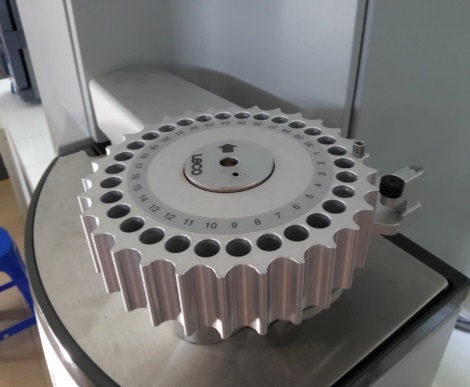


Specification of Forced Draft Blower

| Specification of Force Draft Blower | |
| --- | --- |
| Size | 2” |
| Phase | 1 Phase |
| Voltage | 220 V |
| Cycle | 50/60 |
| Ampere | 1 A |
| R.P.M. | 3000/3600 |

Specification of Suction Blower

| Specification of Suction Blower | | | | | | | | | |
| --- | --- | --- | --- | --- | --- | --- | --- | --- | --- |
| Voltage | Power Input | | Air Flow | Pressure Rise | | Cycle | Poles | | Model |
| 220 V | 530 Watt | | 14.8 m^3^/min | 100 mmAq | | 50 Hz | 2P | | BW-701S |
| Specification of Suction Blower | | | | | | | | | |
| Voltage | | 220 V | | | Cycle | | | 50 Hz | |
| Power Input | | 530 Watt | | | Poles | | | 2P | |
| Air Flow | | 14.8 m^3^/min | | | Model | | | BW-701S | |
| Pressure Rise | | 100 mmAq | | | Manufacturing Date | | | 2006 | |

Specification of Anemometer


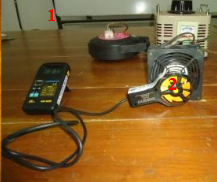


Specification of thermocouple-K

Range temperature : -50^o^ C – 1300^o^ C


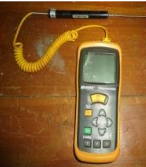


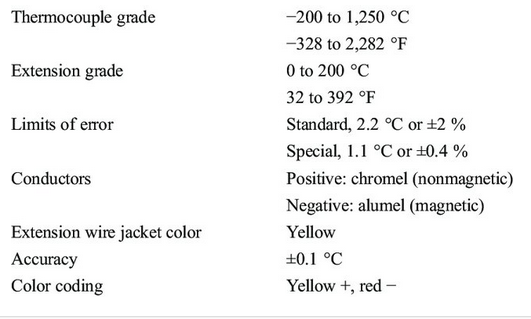


Specification of water pump pompa panasonic GP-129JXK


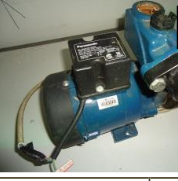


| Kapasitas Maksimum | 30 lt/menit |
| --- | --- |
| Daya Hisap | 9 meter |
| Total Head Maksimum | 30 meter |
| Daya Motor | 125 watt |
| Pipa Hisap | 1 inci (25mm) |
| Pipa Dorong | 1 inci (25mm) |
| Ukuran (P x L x T ) | 206 x 152 x 212 mm |
| Berat | 5.4 kg |

Specification of digital stopwatch


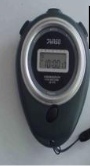


- Square design, plastic construction and LCD display
- Professional sports chronograph digital timer stopwatch show hour, minute, second, AM / PM indicator, month, data, and day of the week
- You can select 12 or 24 hour user with chronograph stopwatches 1/100 second chronograph up to 23 hours, 59 minutes, 59 seconds - Timer stopwatches alarm with 4 minutes snooze
- Powered by one AG13 button cell (included)
- Hourly chime function and alarm function are included
- It comes with a nylon fabric neck strap for easy carrying
- SPLIT / RESET, MODE and START / STOP buttons for convenient operation
- Dial Window Material Type: Plastic
- Dial Display: Digital
- Style: Sport
- Color: Black
- Size(L x W x H): 78 x 63 x 18mm
- Net Weight: 46g

Specification of digital scale CAS-SW 1A CAP 30 kg


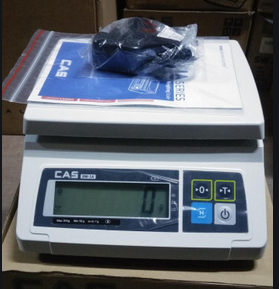


| Max.capacity | 30 kg |
| --- | --- |
| Readibility | 0.2 gr |
| Resolution | /30.000 |
| Display Type | 1: LCD Backlight |
| Operating temperature | -10 C ~ 40 C |
| Power | DC9V Adaptor |
| Battery Life | Dry battery : Alakaline : 800hrs, min 400hrs |
| Platter Size (mm) | 230 (W) x 190 (D) |
| Dimensions (mm) | 260 (W) x 287 (D) x 137 (H) |
| Product weight (kg) | 2.8. |

Specification of digital multimeter Fluke 179 True-RMS


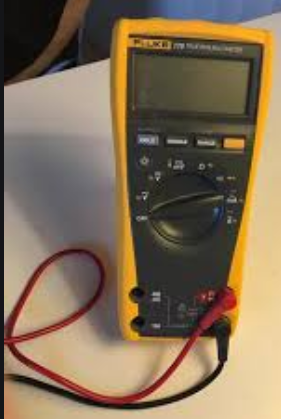


| Voltage DC | Accuracy^1^ |
| --- | --- |
| Operating temperature | -10°C to +50°C |
| Storage temperature | -30°C to +60°C |
| Humidity (without condensation) | 0% – 90% (0°C – 35°C) 0% – 70% (35°C – 50°C) |
